# Variation in Recombination Rate Is Shaped by Domestication and Environmental Conditions in Barley

**DOI:** 10.1093/molbev/msz141

**Published:** 2019-06-18

**Authors:** Steven Dreissig, Martin Mascher, Stefan Heckmann

**Affiliations:** 1 Meiosis Research Group, Leibniz Institute of Plant Genetics and Crop Plant Research (IPK) OT Gatersleben, Seeland, Germany; 2 Domestication Genomics Research Group, Leibniz Institute of Plant Genetics and Crop Plant Research (IPK) OT Gatersleben, Seeland, Germany; 3 German Centre for Integrative Biodiversity Research (iDiv) Halle-Jena-Leipzig, Leipzig, Germany

**Keywords:** recombination landscape, natural variation, climate conditions, domestication

## Abstract

Meiotic recombination generates genetic diversity upon which selection can act. Recombination rates are highly variable between species, populations, individuals, sexes, chromosomes, and chromosomal regions. The underlying mechanisms are controlled at the genetic and epigenetic level and show plasticity toward the environment. Environmental plasticity may be divided into short- and long-term responses. We estimated recombination rates in natural populations of wild barley and domesticated landraces using a population genetics approach. We analyzed recombination landscapes in wild barley and domesticated landraces at high resolution. In wild barley, high recombination rates are found in more interstitial chromosome regions in contrast to distal chromosome regions in domesticated barley. Among subpopulations of wild barley, natural variation in effective recombination rate is correlated with temperature, isothermality, and solar radiation in a nonlinear manner. A positive linear correlation was found between effective recombination rate and annual precipitation. We discuss our findings with respect to how the environment might shape effective recombination rates in natural populations. Higher recombination rates in wild barley populations subjected to specific environmental conditions could be a means to maintain fitness in a strictly inbreeding species.

## Introduction

Meiotic recombination, the exchange of DNA between homologous chromosomes, and the random segregation of chromosomes into gametes are fundamental to eukaryotic reproduction. Novel allelic combinations are generated through recombination upon which selection can act, which facilitates adaptation. Recombination may create novel beneficial combinations of alleles as well as break apart favorable ones (Barton 1995; Charlesworth and Barton 1996; Otto 2009). In addition to recombination and random segregation, different breeding systems are thought to have an impact on maintaining genetic diversity in a population (Jain 1976). Among plants, there are outbreeding species such as *Secale cereale* or *Hordeum bulbosum* showing high levels of heterozygosity, and strictly inbreeding species such as *Triticum aestivum* and *Hordeum vulgare* showing high levels of homozygosity (Johnston et al. 2009; International Barley Genome Sequencing Consortium et al. 2012; Bauer et al. 2017; Mascher et al. 2017; International Wheat Genome Sequencing Consortium et al. 2018). In strictly inbreeding species such as barley (*H. vulgare*) and its wild relative *H. vulgare* spp. *spontaneum* (K. Koch) Thell (Brown et al. 1978), the loss of flexibility may provide short-term advantages coupled with long-term disadvantages (Jain 1976). As a main driver of genetic variation, recombination rates are highly variable at multiple scales, for example, between different species (Auton et al. 2012; Stapley et al. 2017), populations of the same species (Kong et al. 2010; Salomé et al. 2012; Spence JP, Song YS, unpublished data), individuals of the same population (Wang et al. 2012), and sexes (Kong et al. 2010; Kianian et al. 2018). Recombination rates vary even along chromosomes, with elevated recombination rates found near the distal ends of the chromosomes and low recombination rates surrounding the pericentromere in most organisms with large genomes (Stapley et al. 2017; Haenel et al. 2018). At fine scales, recombination tends to be focused in narrow hotspots determined by the zinc finger DNA binding protein PRDM9 in most mammals (Boulton et al. 1997; Oliver et al. 2009; Baudat et al. 2010; Myers et al. 2010). In plants, which lack PRDM9, recombination hotspots are determined by chromatin features and are often found within gene promoters (Choi and Henderson 2015). However, similar to rapidly evolving PRDM9 hotspots in mammals (Boulton et al. 1997; Oliver et al. 2009; Baudat et al. 2010; Myers et al. 2010), different *Theobroma cacao* and rice populations show largely divergent hotspot locations influenced by retrotransposon abundance and genetic divergence (Marand et al. 2019; Schwarzkopf EJ, Motamayor JC, Cornejo OE, unpublished data).

Variation in recombination rate is influenced by genetic, epigenetic, and environmental factors. Genetic divergence and copy number variation of recombination modifiers impact patterns of recombination in plants (Ziolkowski et al. 2015, 2017). Recombination rates are influenced by DNA methylation (Melamed-Bessudo and Levy 2012; Mirouze et al. 2012; Yelina et al. 2012; Habu et al. 2015), histone modifications (Choi et al. 2013; Underwood et al. 2018), and nucleosome occupancy (Choi et al. 2018). Environmental effects on patterns of recombination reveal no clear consensus owing to differences between species and experimental systems. For example, the relationship between temperature and recombination was found to resemble an U-shaped curve, with elevated recombination rates found at low and high temperatures (Plough 1917; Plough 1921; Lloyd et al. 2018; Modliszewski et al. 2018), or a reverse U-shaped curve (Yanney Wilson 1959). Additionally, positive or negative linear correlations were described (Bomblies et al. 2015; Jackson et al. 2015; Phillips et al. 2015). In Drosophila, desiccation, hypoxia, and hyperoxia affect recombination rates, indicating fitness-related plasticity (Aggarwal et al. 2015; Aggarwal DD, Rybnikov SR, Cohen I, Rashkovetsky E, Michalak P, Korol AB, unpublished data). Recombination rates are further influenced by nutritional status or pathogen attack (Law 1963; Kovalchuk et al. 2003; Andronic 2012; Fuchs et al. 2018; Rey et al. 2018). As mentioned by Bomblies et al. (2015), these various correlations bring to mind the question of whether environmental factors act independently or in concert. However, most studies focused on the effect of extreme environmental stress over a single generation, with exceptions in Drosophila, where short breeding cycles allowed observations over a few hundred generations (Law 1963; Kovalchuk et al. 2003; Aggarwal et al. 2015; Phillips et al. 2015; Lloyd et al. 2018).

In the present study, we used a population genetic approach to estimate effective recombination rates in a strictly inbreeding grass species, *H. vulgare*, and asked whether natural variation in recombination rates is associated with present (1970–2000) and past (6,000–22,000 years before present [BP]) environmental conditions. We find strong similarities between the recombination landscape of wild and domesticated barley based on population genetic data. However, fine-scale differences regarding the physical distribution of recombination events are detected. Finally, we observe natural variation in recombination rate among subpopulations of wild barley and find strong associations with temperature, isothermality, solar radiation, and precipitation.

## Results and Discussion

### Recombination Landscapes Are Highly Conserved between Wild and Domesticated Barley

The physical distribution and frequency of recombination events, that is, recombination landscape, play a role in plant adaptation as some genes are more likely to recombine than others. The barley genome is highly compartmentalized, with for example, disease resistance genes located in highly recombining distal regions of the chromosomes and genes involved in photosynthesis in low-recombining interstitial regions (Mascher et al. 2017). Previous characterizations of the recombination landscape of barley focused on domesticated barley (Künzel et al. 2000; Higgins et al. 2012; Phillips et al. 2012, 2015; Dreissig et al. 2015, 2017), except for cytological studies revealing a slightly higher number of chiasmata in domesticated barley than in wild barley (Ross-Ibarra 2004). Here, we asked whether the fine-scale physical distribution of recombination events differs between domesticated and wild barleys. We hypothesized different recombination landscapes might be a consequence of adaptation to different environments, for example, natural habitats versus post-Neolithic farming.

**Fig. 1.**
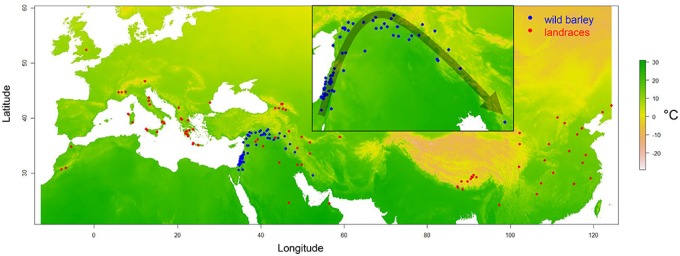
Origin of wild barley and landrace accessions. Collection sites of wild barley accessions (blue) and barley landraces (red) are shown. Colouring represents annual mean temperature under present conditions (°C). Within the inlet, which is zoomed in on the Fertile Crescent, the black arrow indicates the direction in which wild barley sub-populations were sampled.

**Fig. 2.**
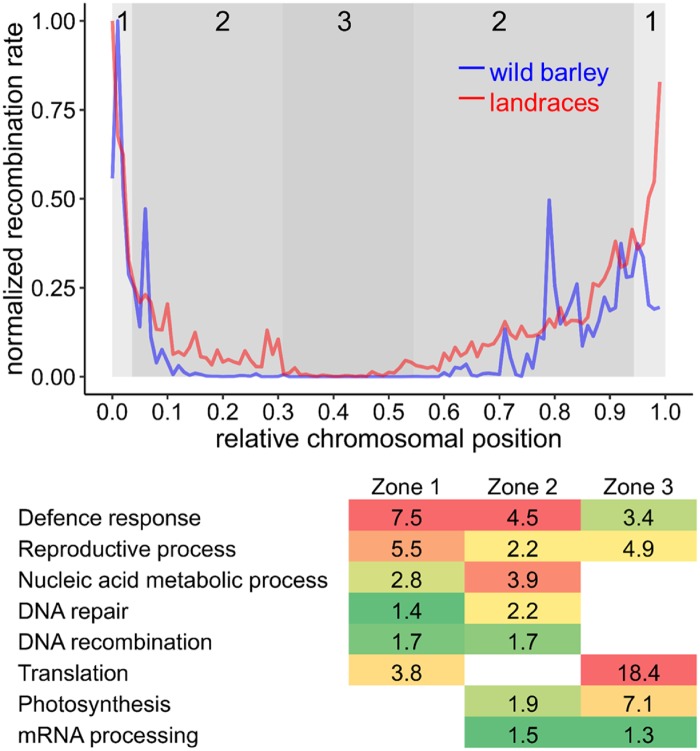
Comparison of recombination landscapes between wild barley and domesticated landraces. Normalized recombination rate (0 = lowest value, 1 = highest value within a population) of wild barley (blue) and domesticated landraces (red) along relative chromosomal positions (0 = distal end of the short arm, 1 = distal end of the long arm) derived from the average of all seven chromosomes. On the short arm, highest values in both wild and domesticated barley overlap within the distal tip (5%) of the chromosome. On the long arm, highest values of wild barley reside within 80–90% of chromosome length, whereas highest values of domesticated barley reside within the distal tip (90–100% chromosome length). Enrichment of Gene Ontology (GO) terms in genomic compartments are adopted from Mascher et al. (2017). Coloured rectangles indicate −log_10_-transformed *P-*values from 1.3 (green) to 18.4 (red).

In order to estimate recombination rates in wild barley (*H. vulgare* spp. *spontaneum* (K. Koch) Thell), we applied coalescent theory to a single nucleotide polymorphism (SNP) data set comprising 26,417 positions in a natural population of 74 geo-referenced accessions (fig. 1) (Milner et al. 2019). The Interval program from the LDhat package was used to estimate the population-scaled recombination rate (ρ) along the seven chromosomes of barley. We validated ancestral recombination rates estimated from population genetic data by comparing them to experimental measurements obtained through pollen nuclei sequencing of an F_1_ hybrid between two modern barley cultivars (Dreissig et al. 2017). We observed a positive correlation of 0.81 between ancestral and experimental recombination landscape across relative chromosomal intervals of 0.01 (% of total chromosome length, *P *=* *1.48* *×* *10^−24^). These correlations are comparable to previous work in Arabidopsis (Choi et al. 2013) and wheat (Darrier et al. 2017), showing that coalescent-based methods provide reliable estimates of recombination landscapes. Next, we attempted to compare wild barley and domesticated landraces in order to test if the domestication process had an impact on their respective recombination landscapes. We estimated the population-scaled recombination rate in barley landraces using a large SNP data set comprising 26,334 SNPs and 100 randomly selected geo-referenced barley landraces (fig. 1, Milner et al. 2019). For both wild barley and landraces, ρ was summed over relative chromosomal intervals and averaged across all seven chromosomes. Based on Spearman’s rank correlation, the two recombination landscapes are highly similar (*r *=* *0.91, *P *=* *2.08* *×* *10^−39^). Elevated recombination rates are strictly confined to distal chromosomal regions, leaving about 80% of the chromosomes nearly devoid of recombination (fig. 2). In both, the extent of the recombining region is smaller on the short arm and greater on the long arm of all chromosomes. At the fine-scale, however, differences became visible. On the long arm, elevated recombination rates are detected in more interstitial regions in wild barley (fig. 2, 80–90% relative chromosome length), which is most pronounced on chromosome 2H, 3H, 5H, 6H, and 7H (supplementary fig. 2, Supplementary Material online). In domesticated landraces, elevated recombination rates are more distally confined on the long arm (90–100% relative chromosome length), with no striking difference between chromosomes except for 7H (supplementary fig. 2, Supplementary Material online). On the short arm, however, this does not seem to be the case, as elevated recombination rates are strictly confined to the first 5% of the chromosome in both groups.

Wild barley was estimated to have diverged from its most recent common ancestor approximately 4 Ma (Brassac and Blattner 2015), and domestication began approximately 10,000 years ago (Badr et al. 2000). By comparing the recombination landscape of wild barley with that of domesticated barley landraces, we show that recombination landscapes are highly conserved throughout domestication. Our data provide evidence for a strict separation between chromosomal regions permissive for recombination and chromosomal regions suppressive for recombination, even in long-term ancestral recombination data. Previous work demonstrated meiotic recombination is largely suppressed in heterochromatic regions enriched in CG, CHG, and CHH DNA methylation (Melamed-Bessudo and Levy 2012; Mirouze et al. 2012; Yelina et al. 2012), and histone modifications such as H3K27me3, H3K9me3, H3K27me1, and H3K9me2 (Aliyeva-Schnorr et al. 2015; Baker et al. 2015). A possible explanation for our observations could be that the chromatin environment suppressive for recombination is highly conserved across evolutionary time-scales. At the fine-scale, however, elevated recombination rates are shifted toward more distal regions on the long arm of the chromosomes in domesticated barley. Distal and interstitial regions show different gene contexts, with distal regions enriched for defense response genes and interstitial regions rather enriched for genes involved in basic cellular processes, such as nucleic acid metabolism, DNA repair, photosynthesis, and mRNA processing (fig. 2). As a consequence of this compartmentalization, differences in recombination rate might be caused by selection for elevated recombination rates in regions harboring defense response genes throughout barley’s domestication, as recombination hotspots tend to be found near disease resistance genes (Serra et al. 2018). Wild barley, which is not exposed to high pathogen pressure and does not show strong selection on resistance genes (Stukenbrock and McDonald 2008; Ma et al. 2019), may therefore show a different ancestral recombination landscape.

### Natural Variation in Recombination Rate

Recombination rates are highly variable at multiple scales, such as along chromosomes, sexes, individuals, populations, and species (Stapley et al. 2017). In a strictly inbreeding species such as barley (Brown et al. 1978), recombination may be under selection to counterbalance inbreeding depressions and maintain fitness (Charlesworth et al. 1977). Previous studies have shown increased chiasmata frequency in inbreeding plants (Stebbins 1950; Rees and Ahmad 1963; Zarchi et al. 1972; Gibbs et al. 1975). In this study, we asked whether recombination rates differ among natural populations of wild barley.

To analyze variation in recombination rate within a wild barley population, subpopulations needed to be defined for which recombination rates could be estimated. We first performed a principal component analysis (PCA) to test for population structure. The first two principal components explained 8.28% of the observed variance and revealed a continuous gradient along the Fertile Crescent, resembling its shape in the PCA space (supplementary fig. 3*A*, Supplementary Material online). We analyzed population admixture using sNMF (Frichot et al. 2014) with the number of ancestral populations (*K*) ranging from 1 to 20. As *K* increased, the cross-entropy criterion decreased and no local minimum was reached (supplementary fig. 3*B* and *C*, Supplementary Material online). This suggested a continuous genetic gradient along the Fertile Crescent, which is supported by the absence of major geographic obstacles.

**Fig. 3.**
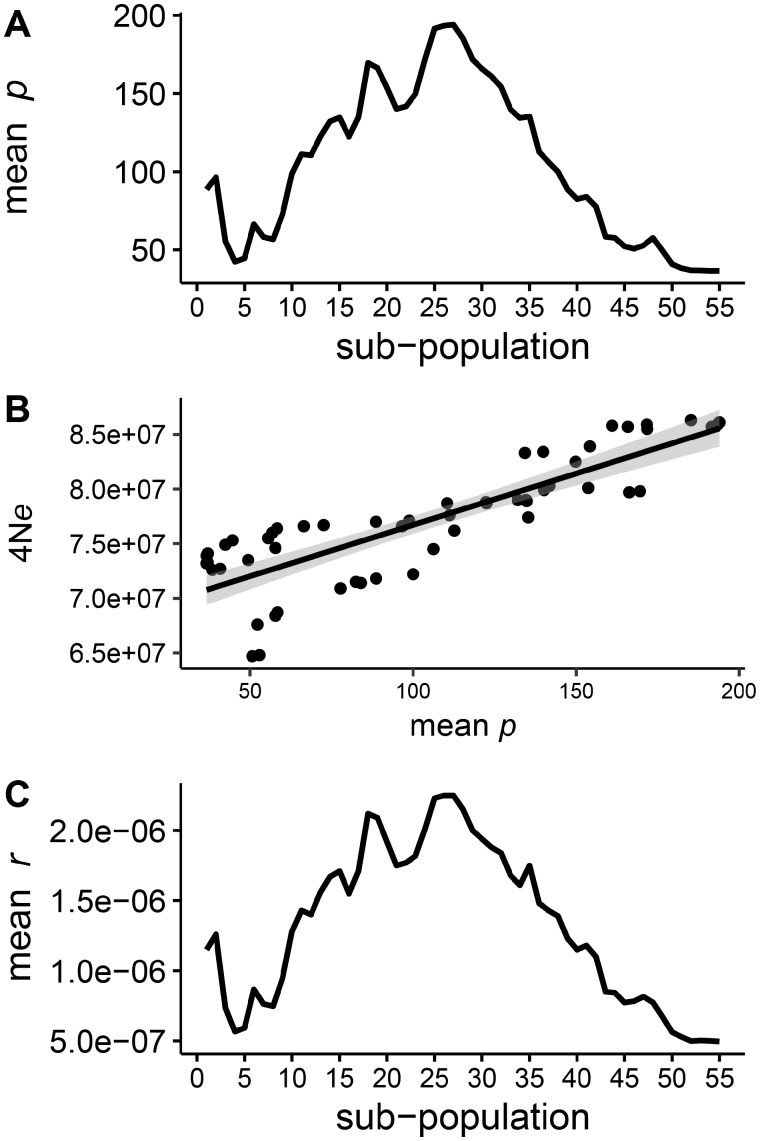
Subpopulation analysis of ρ, 4*N_e_*, and *r* in geo-referenced wild barley accessions. Seventy-four geo-referenced wild barley accessions were divided into 55 sub-populations of 20 accessions per sub-population according to a sliding window approach with a step size of 1 accession. Sliding windows are moved along the geographical distribution of wild barley across the Fertile Crescent. (*A*) Estimation of genome-wide mean population-scaled recombination rate (ρ). (*B*) Correlation between effective population size (4*N_e_*), based on estimates of Watterson’s theta (*theta*_W_) and an assumed mutation rate (*mu*) of 3.5* *×* *10^−9^, and population-scaled recombination rate (ρ). (*C*) Genome-wide mean effective recombination rate (*r_e_*) corrected for differences in effective population size.

Since it was not feasible to define subpopulations based on ancestry coefficients, we instead defined overlapping subpopulations based on the geographical distribution of wild barley in agreement with their distribution in the PCA space. Subpopulations were defined following a sliding window approach comprising 20 accessions per window with a step size of 1 accession. Sliding windows were moved along the geographical distribution of wild barley in the Fertile Crescent (fig. 1, Russell et al. 2014, 2016). In total, population-scaled recombination rates (ρ) were estimated in 55 subpopulations and averaged across all 7 chromosomes. Our analysis revealed substantial variation among subpopulations (fig. 3*A*). Importantly, similar trends were observed between individual chromosomes (supplementary fig. 4, Supplementary Material online). For example, the lowest and highest genome-wide average ρ varied by a factor of 5.3. Since ρ is affected by effective population size (ρ = 4*N_e_ *×* r*), we estimated 4*N_e_* based on nucleotide diversity (*theta*_W_, Watterson’s theta) in each subpopulation and an assumed mutation rate (*mu*) of 3.5* *×* *10^−9^ per bp per year (Lin et al. 2002). Effective population size varied between subpopulations by a factor of 1.33 and was positively correlated with ρ (fig. 3*B*, *r *=* *0.79, *P *=* *4.85* *×* *10^−13^). We used the estimates of 4*N_e_* to obtain the effective recombination rate per generation in each subpopulation (*r_e_* = ρ/4*N_e_*). After correcting for differences in effective population size, variation in effective recombination rate (*r_e_*) remained largely unchanged, with the lowest and highest genome-wide average *r_e_* varying by a factor of 4.54 (fig. 3*C*, Kruskal–Wallis test, *P *<* *2.2* *×* *10^−16^). Therefore, variation in the population-scaled recombination rate seemed unlikely to be entirely caused by differences in effective population size. However, it cannot be excluded that different populations may experience different mutation rates. We also tested whether the observed pattern is explained by geographical distance within subpopulations, which may result in higher genetic diversity in subpopulations spanning wider geographical ranges (Owuor et al. 1997; Hübner et al. 2009; Russell et al. 2014) affecting estimates of the population-scaled recombination rate. For each subpopulation, we calculated the longitudinal, latitudinal, and altitudinal range as a measure of geographical distance. For example, almost the whole range of recombination rate values was found twice over distinct geographical ranges (e.g., 50–70 and 300–400 km, supplementary fig. 5, Supplementary Material online). A low number of haplotypes in narrow populations, and a lack of contact in extremely dispersed populations could cause low recombination rates in extremely narrow or dispersed populations. On the other hand, the absence of a linear association and our observation of the full range of recombination rate values over similar geographical ranges suggest that geographical distance does not primarily explain variation in the effective recombination rate. Finally, estimates of effective recombination rates may be influenced by selection. Based on previous work showing that wild barley is not subjected to strong selection and rather found in a state of random genetic drift (Russell et al. 2016; Milner et al. 2019), we conclude the observed differences are unlikely to be caused by patterns of selection. Taken together, effective recombination rates are potentially affected by a multitude of population genetic factors, as well as actual differences in meiotic recombination rate.

### Environmental Factors Shape Effective Recombination Rate in Natural Populations

There is a large body of experimental evidence showing correlations between recombination rates and environmental conditions. Particularly, the effect of temperature on meiotic recombination was studied in a number of experimental systems, such as Drosophila, Arabidopsis, barley, and other plants (Dowrick 1957; Mange 1968; Zhuchenko et al. 1985; Jackson et al. 2015; Phillips et al. 2015; Lloyd et al. 2018; Modliszewski et al. 2018). However, as mentioned by Lloyd et al. (2018), an interesting question is whether these observations are reflective of what occurs in natural populations. Therefore, we sought to explore the relationship between effective recombination rate and environmental conditions in natural populations.

**Fig. 4.**
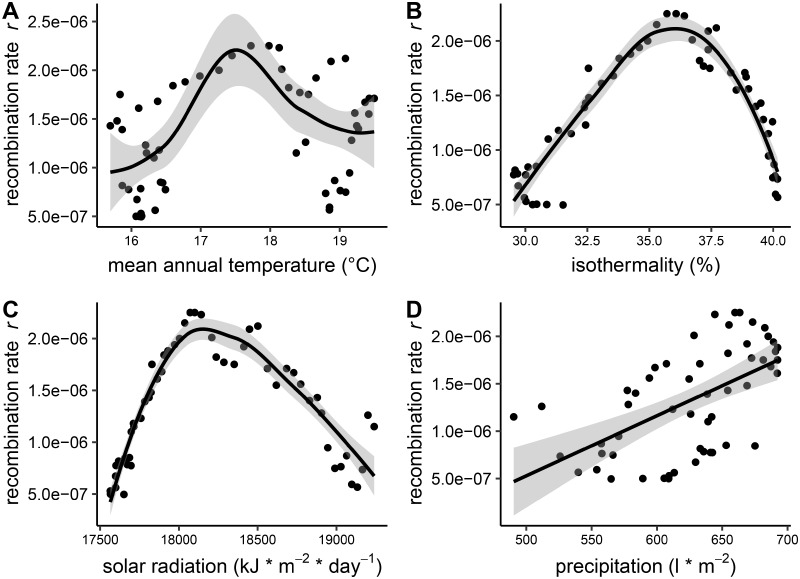
Correlation between recombination rate and environmental variables. Recombination rate estimated in 55 subpopulations of wild barley is plotted against environmental variables. The black line represents a smoothed curve over the data and the gray area represents the 95% confidence interval of the smoothed curve. (*A*) Relationship between recombination rate and annual mean temperature under Mid Holocene conditions. (*B*) Reverse U-shaped relationship between recombination rate and isothermality under Mid Holocene conditions. (*C*) Reverse U-shaped relationship between recombination rate and annual mean solar radiation under present conditions. (*D*) Correlation between recombination rate and annual precipitation under Mid Holocene conditions.

To address this question in natural populations of wild barley, we extracted annual mean temperature values for 74 geo-referenced wild barley accessions under present (1970–2000), Mid Holocene (MH, about 6,000 years BP), and Last Glacial Maximum (LGM, about 22,000 years BP) conditions. After the LGM and throughout the MH, wild barley showed a range expansion across the Fertile Crescent reflecting its current geographical distribution (Russell et al. 2014). We therefore focused on environmental conditions during the MH. Plotting recombination rate against annual mean temperature revealed a nonlinear relationship between temperature and recombination rate (fig. 4*A*). Across an annual mean temperature range from 15.6 to 19.5 °C, recombination rate was lower in subpopulations at either end of the scale and higher over the intermediate range, showing a reverse U-shaped curve. The same trend was observed by correlating recombination with different temperature conditions, that is, present conditions, MH conditions, and LGM conditions (supplementary figs. 6 and 7, Supplementary Material online). Considering the contribution of meiotic recombination to the effective recombination rate, it is important to consider the timing of meiosis. Meiosis usually takes place in spring under temperatures, which may be different to the annual mean. We therefore used present climate data to test if the trend observed for annual temperature is similar to that observed for approximate spring temperatures, which we considered the mean of March and April. A significant positive correlation indicated that annual mean temperature values reveal a similar trend as spring temperatures ranging from 12 to 16 °C (Pearson’s *r *=* *0.978, *P *=* *1.28* *×* *10^−37^).

Interestingly, variation in recombination rate was best explained by isothermality (fig. 4*B*), which describes temperature variability based on the day-to-night temperature range relative to the summer-to-winter temperature range (i.e., higher values indicating larger temperature variation and vice versa). We observed a reverse U-shaped curve, showing higher recombination rates across an intermediate isothermality range and lower recombination rates at either end of the scale. To test for a systematic bias in our sliding window approach, we performed the same analysis on a set of 55 randomized subpopulations, that is, randomly grouped accessions contrary to grouped according to geographical distribution, focusing on one representative chromosome. When subpopulations were chosen randomly, no correlation with temperature or isothermality was found (MH annual mean temperature: *P *=* *0.11; MH isothermality: *P *=* *0.11; supplementary fig. 8, Supplementary Material online).

In addition to temperature, we also observed a nonlinear relationship between recombination rate and annual solar radiation (fig. 4*C*). A positive linear relationship was observed with annual precipitation across the three different climate conditions (fig. 4*D*, present: *r *=* *0.298, *P *=* *0.027; MH: *r *=* *0.572, *P *=* *5.1* *×* *10^−6^; LGM: *r *=* *0.765, *P *=* *1.1* *×* *10^−11^). Across time, from past (LGM) to present conditions, annual precipitation generally decreased in the Fertile Crescent. Interestingly, higher precipitation under LGM conditions appears to be better suited to explain differences in recombination rate. This is in agreement with a positive correlation between outcrossing rate and annual precipitation (Abdel-Ghani et al. 2004), which also results in higher effective recombination rates (Nordborg 2000). In barley, self-fertilization occurs while the spike is still enclosed in the flag leaf sheath (Alqudah and Schnurbusch 2017), which results in a high inbreeding coefficient in our data (*F *=* *0.978, CV = 0.02%). Therefore, although outcrossing does play a role, we conclude it is likely to have a minor effect in strictly inbreeding barley.

**Fig. 5.**
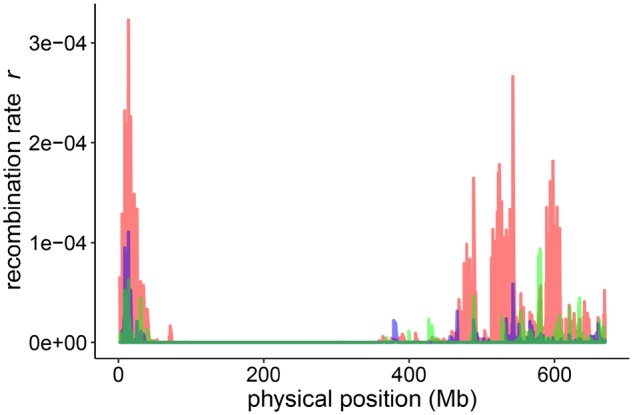
Recombination landscapes of different sub-populations. Recombination rates of different sub-populations (red = #27, blue = #4, green = #55) along the physical length of chromosome 5H. These three subpopulation were selected to represent the observed minima and maxima among all subpopulations. Variation in recombination rate is strictly confined to distal regions of the chromosome and no variation is observed in the low-recombining pericentromeric region.

The differences in effective recombination rate between subpopulations were strictly confined to distal regions of the chromosome and no difference was observed in interstitial regions (fig. 5). Considering the contribution of the meiotic recombination rate to the effective recombination rate, it is tempting to speculate on the molecular mechanisms leading to an increase in physically confined regions of the chromosomes. In plants, Drosophila, and yeast, temperature was shown to affect the frequency and distribution of class I crossover as well as meiotic chromosome axis and synaptonemal complex length (Börner et al. 2004; Higgins et al. 2012; Aggarwal et al. 2015; Phillips et al. 2015; Lloyd et al. 2018; Modliszewski et al. 2018). This implies a mechanistic role of some proteins involved in meiotic chromosome axis, synaptonemal complex, and/or CO formation in mediating temperature-dependent plasticity of the recombination landscape which might in part be of biophysical origin as various proteins involved in these processes and their activity are temperature sensitive (Morgan et al. 2017). In addition, UV-radiation and the nutritional state of the plant affect the formation of DNA double strand breaks and meiotic recombination (Grant 1952; Griffing and Langridge 1963; Ries et al. 2000; Knoll et al. 2014; Aggarwal et al. 2015; Mercier et al. 2015; Si et al. 2015; Martín et al. 2017; Rey et al. 2018; Aggarwal DD, Rybnikov SR, Cohen I, Rashkovetsky E, Michalak P, Korol AB, unpublished data).

Our observations suggest that effective recombination rates are higher in populations subjected to intermediate annual temperature, isothermality, and solar radiation rather than extremes. This seems to be contradictory of what was observed in experimental studies in Arabidopsis and barley (Phillips et al. 2015; Lloyd et al. 2018; Modliszewski et al. 2018), where temperature extremes were associated with increased recombination rate. However, key differences between our observations and experimental studies are the number of generations analyzed, the multitude of environmental conditions considered, and population genetic factors. Effective recombination rates estimated by patterns of linkage disequilibrium (LD) are the result of thousands of rounds of recombination and patterns of selection, outcrossing, and ancestral admixture. On the other hand, experimental studies are often limited to one generation owing to the time it takes to cultivate plants. We therefore think of our observations as long-term effective recombination rates, which may differ from short-term responses of the recombination machinery to environmental stress. These differences between long- and short-term effects might be influenced by different alleles of meiotic regulators, leading to differences in recombination rates (Sidhu et al. 2017; Ziolkowski et al. 2017). However, since key meiotic regulators generally show high sequence conservation (Villeneuve and Hillers 2001; Sidhu et al. 2017), our observations may be explained by meiotic plasticity toward the environment. Our data do not enable us to dissect the precise environmental conditions every population/individual faced during each meiotic cycle over time within the Fertile Crescent. However, they allow us to describe broad environmental effects on the recombination landscape of natural populations. For a strictly inbreeding species such as barley, the observed pattern can be interpreted as a measure to balance the loss of fitness caused by inbreeding. Interestingly, Morrell et al. (2005) observed surprisingly low levels of LD in wild barley, comparable to that of *Zea mays*, an outbreeding species. Our data provide evidence for high effective recombination rates under specific environmental conditions, which could explain the rapid LD decay observed by Morrell et al. (2005).

Taken together, our observations imply differences in how effective recombination rates are correlated with environmental conditions over long periods of time versus short-term responses of the recombination machinery to environmental stress. Under natural conditions, plant populations are subjected to varying environmental conditions, which are tightly linked, such as temperature, light, and precipitation. In controlled experiments, however, often only a single parameter of interest is changed in order to study its effects. Our observations suggest an interplay between temperature, light, and precipitation shaping variation in effective recombination rate in wild barley. A maximum effective recombination rate under intermediate temperature and light, as well as high precipitation may be interpreted as a means of generating genetic diversity upon which selection can act (Presgraves 2005). In a strictly inbreeding species, this might be a mechanism to counteract the negative effects of inbreeding and maintain fitness.

## Materials and Methods

### Estimation of Population-Scaled Recombination Rate Using LDhat

In the present study, we used genotyping-by-sequencing (GBS) data with imputed SNP calls of 74 geo-referenced wild barley accessions (*H. vulgare* spp. *spontaneum* (K. Koch) Thell) and 100 domesticated barley landraces (supplementary file 1, Supplementary Material online) (Milner et al. 2019). Imputation of missing genotypes was performed with FILLIN (Swarts et al. 2014). SNPs with (1) less than 95% missing data, (2) less than 2% heterozygous calls, and (3) a minor allele count ≧10 in a set of 1,140 wild barleys were used as input for imputation (data stored on e!DAL [Arend et al. 2014] http://dx.doi.org/10.5447/IPK/2019/3). The 74 wild barley accessions were chosen out of a total of 1,140 accessions based on the availability of exact geographical coordinates of their collection sites. Accessions with identical collection coordinates were removed to avoid analyzing potential duplications. Barley landraces were randomly chosen out of a total of 19,778, considering only those with exact geographical coordinates available. We selected physically mapped positions in the barley reference genome (Mascher et al. 2017) with <10% missing data, >10% minor allele frequency. Since barley is a strictly inbreeding species, heterozygous SNPs were removed and the data were treated as haploid and phased (Brown et al. 1978). We used the Interval program from the LDhat package (Hudson 2001; McVean et al. 2004; Auton and McVean 2007; Choi et al. 2013) to estimate the population-scaled recombination rate (ρ/kb, ρ = 4*N_e_ *×* r_e_*, where *N_e_* is the effective population size and *r_e_* is the effective recombination rate per generation in a population) between pairs of SNPs. In total, 26,417 SNPs out of 689,178 imputed SNPs were used for wild barley with an average resolution of 180.85 kb (supplementary fig. 1, Supplementary Material online). For barley landraces, a total of 26,334 SNPs out of 306,049 imputed SNPs was used with an average resolution of 174.01 kb (supplementary fig. 1, Supplementary Material online). The Interval program was run in 60,000,000 iterations, sampling every 5,000 updates, with a block penalty of 5, and using a population-scaled mutation rate per site of *theta* (*theta* = 4*N_e_ *×* mu*) = 0.01. To obtain a reproducible output from the Interval program, different iteration numbers (10,000,000 vs. 60,000,000), block penalties (5, 10, 15, 20), and *theta* values (0.1, 0.01, 0.001) were tested and selected based on visual comparison of multiple runs. The Stat program from the LDhat package was used to summarize ρ/kb values and the first 25,000 iterations were discarded as burn in.

In order to compare recombination rate estimates inferred from population genetic data to experimental measurements based on pollen nuclei sequencing (Dreissig et al. 2017), ρ/kb was converted to ρ by multiplying by interval width (kb). All chromosomes were partitioned into relative intervals of 0.01% and ρ as well as experimental measurements (cM/Mb) were summed over the same relative intervals. The average of all chromosomes was calculated across the relative intervals to obtain a genome-wide overview. In order to compare recombination landscapes regarding the distribution of maxima and minima, ρ values were normalized within each group by dividing by the maximum value to obtain the normalized recombination rate. Spearman’s rank correlation between population-scaled recombination rate and experimental recombination rate was calculated to test if the observed distributions differ and Student’s *t*-tests were performed to infer statistical significance.

### Analysis of Population Structure

PCA was performed using all SNPs employing the snpgdsPCA() function of the SNPrelate package in R (R Core Team, https://www.r-project.org/contributors.html; last accessed June 18, 2019) which implements the FastPCA algorithm of Galinsky et al. (2016). Partitioning of wild barley accessions into *K* ancestral groups and estimation of individual ancestry coefficients were performed with sNMF (Frichot et al. 2014) using all SNPs. The sNMF algorithm was run for *K* values between 1 and 20 to identify the optimal *K* value using the cross-entropy criterion. We used *K* = 20 to estimate individual ancestry coefficients. Ancestry coefficients were averaged across 15 replications using CLUMPP (Jakobsson and Rosenberg 2007) applying the LargeKGreedy algorithm with 500 permutations.

### Subpopulation Analysis in Sliding Windows

To analyze natural variation in the population-scaled recombination rate within the 74 geo-referenced wild barley accessions, we divided the entire wild barley population into 55 subpopulations of 20 accessions per group according to a sliding window approach with a difference of 1 accession between subpopulations. Sliding windows were selected according to the geographical distribution of wild barley across the Fertile Crescent, starting from the south of Israel, moving further north across the Lebanon to the north-western part of Syria and southern Turkey, further east to the north-eastern part of Syria and south-eastern Turkey, and further south-east across the northern part of Iraq and western part of Iran (fig. 1, Russell et al. 2014). ρ/kb was estimated for each subpopulation using the Interval program with identical parameters as mentioned above.

To account for potential differences in 4*N_e_* between subpopulations, we estimated 4*N_e_* by estimating nucleotide diversity (*theta*_W_, Watterson’s theta) in each subpopulation and assuming a mutation rate per bp (*mu*) of 3.5* *×* *10^−9^ which was previously described for wild barley (Lin et al. 2002). We estimated *theta*_W_ using the Convert program of the LDhat package and used the genome-wide average *theta*_W_ value of each subpopulation to estimate the effective size of each subpopulation. Nucleotide diversity (*theta*_W_) was divided by mutation rate per bp (*mu*) to estimate 4*N_e_* (4*N_e_* = *theta*_W_/*mu*). We then used the estimates of 4*N_e_* to calculate the mean effective recombination rate per generation (*r_e_*) for each subpopulation (*r_e_* = ρ/4*N_e_*). Accessions were also randomly assigned to subpopulations and analyzed in the same way to subsequently test for a systematic bias.

The inbreeding coefficient (F) was calculated in each subpopulation using the –het function of vcftools including all SNPs (Danecek et al. 2011).

### Climate Data and Correlation with Recombination Rate

Climate data were obtained from the global climate database (worldclim.org). Bioclimatic variables, including annual mean temperature (°C), isothermality (%), and annual precipitation (mm) were extracted for present conditions (1970–2000), MH conditions (about 6000 years BP), and LGM conditions (about 22,000 years BP) from the CMIP5 MultiModel Ensemble data set (MPI-ESM-P) from the 30 arc-second and 2.5 arc-minute rasters. Solar radiation values (kJ* *×* *m^−2^* *×* *day^−1^) were extracted for present conditions from 30 arc-second (0.86 km^2^ at the equator) and 2.5 arc-minute (21.6 km^2^ at the equator) rasters. We focused on the above mentioned bioclimatic variables because of the extensively studied effects of temperature, precipitation, and solar radiation on meiosis (see Results and Discussion). To estimate spring temperature, we calculated the average of March and April under present conditions, since no monthly climate data are available under MH or LGM conditions. Additionally, elevation data from the NASA Shuttle Radar Topographic Mission (SRTM) was obtained from the CGIAR Consortium for Spatial Information (http://srtm.csi.cgiar.org/) at 90 m resolution.

We used the “extract” function of the “raster” package of the R statistical environment (R Core Team, https://www.r-project.org/contributors.html) to extract the respective bioclimatic values at the locations of the geo-referenced barley accessions. All values were extracted in a 5 km radius using the “buffer” function and averaged using the “fun=mean” function of the “raster” package.

For each subpopulation, we calculated mean temperature, mean isothermality, mean precipitation, mean solar radiation, and mean elevation. For linear relationships (e.g., precipitation), we calculated Pearson’s correlation coefficient between effective recombination rate (*r_e_*) and bioclimatic variables in each subpopulation. Student’s *t*-tests were performed to infer statistical significance. In case of nonlinear relationships (e.g., annual mean temperature, isothermality, solar radiation), a Kruskal–Wallis test was performed to compare between subpopulations.

## Supplementary Material

Supplementary data are available at *Molecular Biology and Evolution* online.

## Supplementary Material

msz141_Supplementary_Data
