# Supplementary material for: Variation in Recombination Rate Is Shaped by Domestication and Environmental Conditions in Barley
Source: Mol Biol Evol. 2019 Jun 18;36(9):2029–39. doi: 10.1093/molbev/msz141 (PMC6736446; doi:10.1093/molbev/msz141)
Supplement: msz141_Supplementary_Data [file msz141_supplementary_data.zip › Supplementary_Figures.pdf]

Supplementary Fig. 1. SNP density in wild barley and landraces

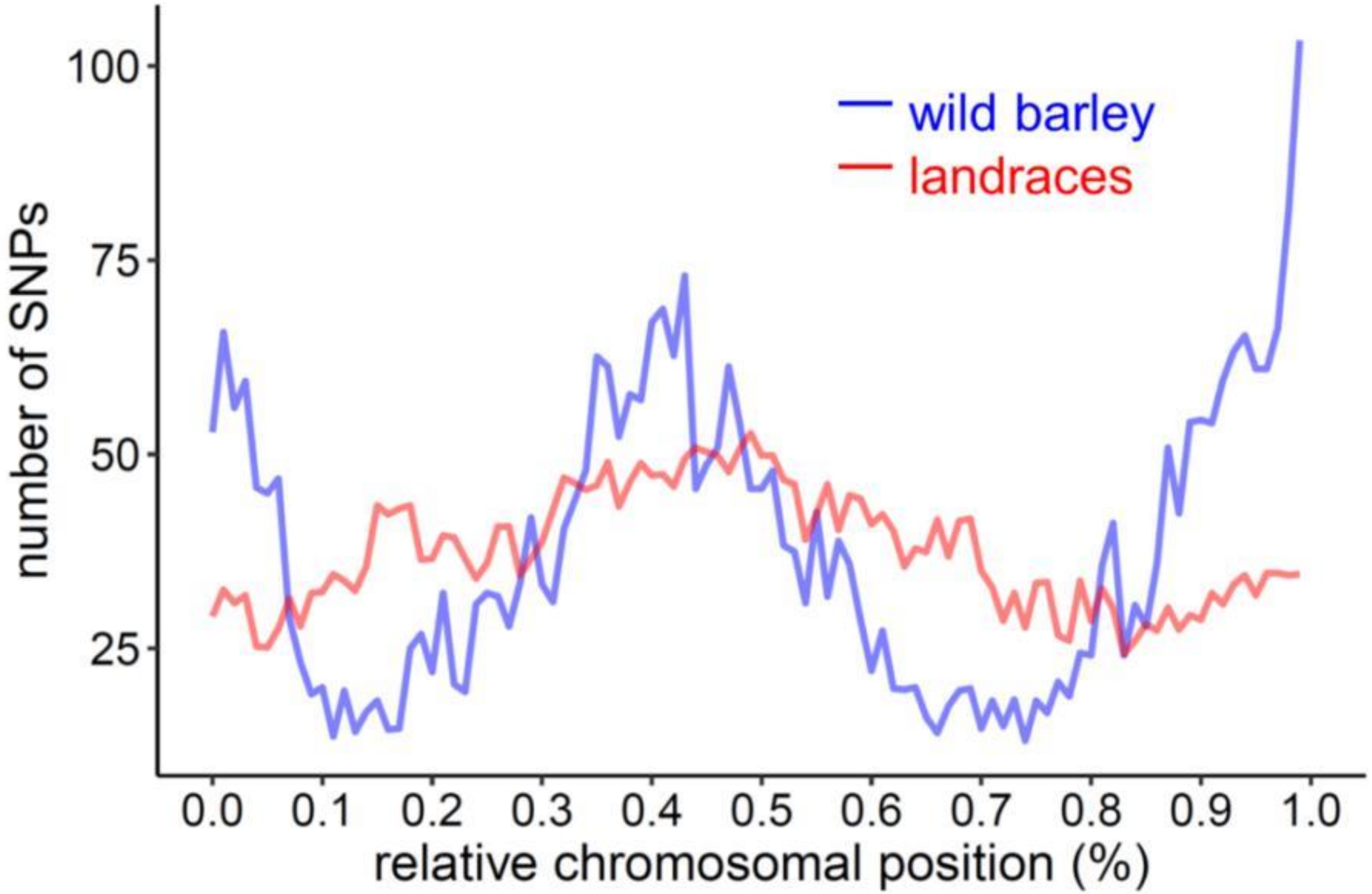

The number of SNPs (blue = wild barley, red = landraces) was counted in relative chromosomal intervals of 0.1% and averaged over all 7 chromosomes.

Supplementary Fig. 2. Normalized recombination rate of wild barley and landraces.

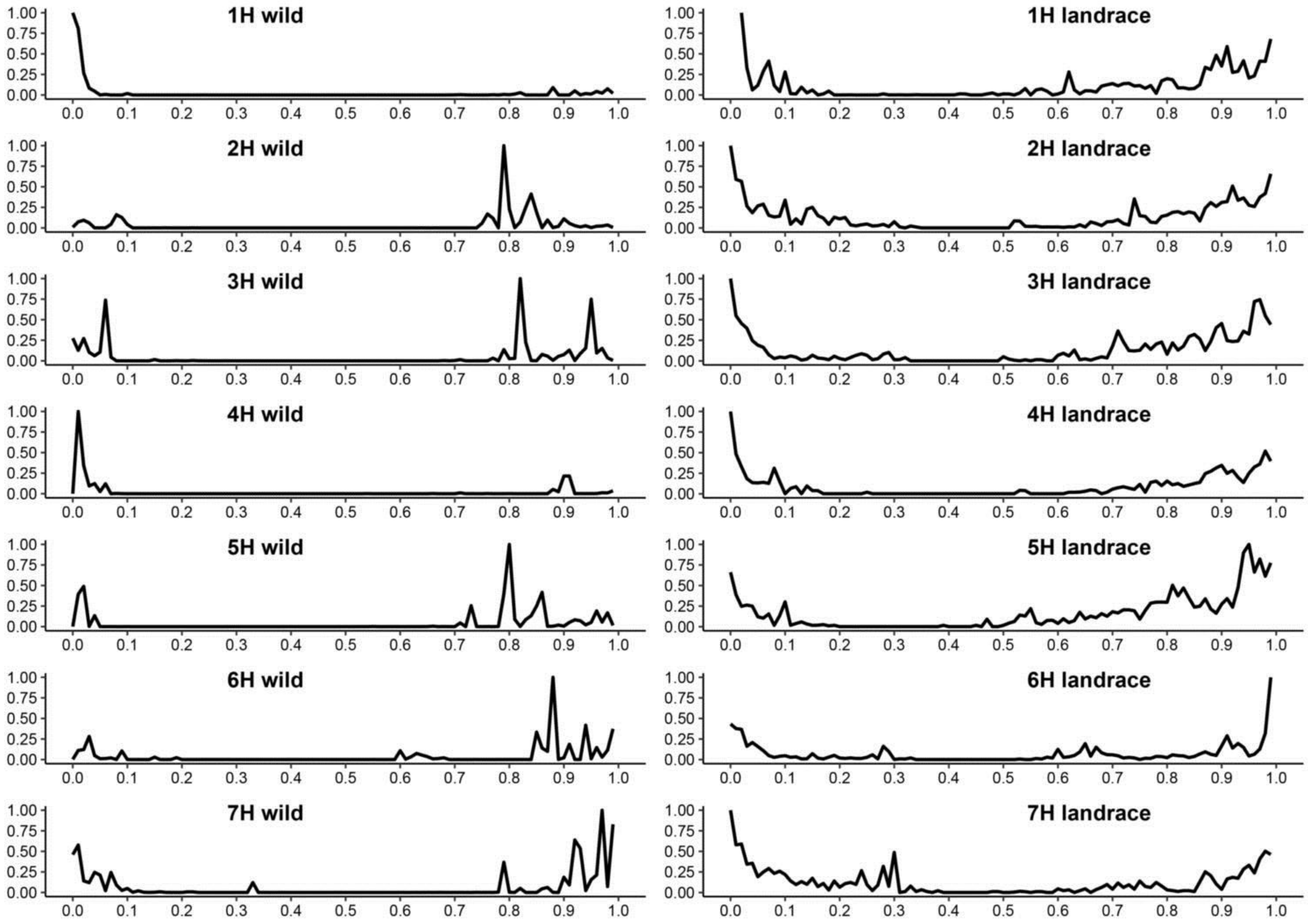

Normalized recombination rate (0 = lowest values, 1 = highest value within a population) is shown on the y axis. Relative chromosomal position (0 = distal end of the short arm, 1 = distal end of the long arm) is shown on the x axis.

Supplementary Fig. 3. Analysis of population structure in wild barley.

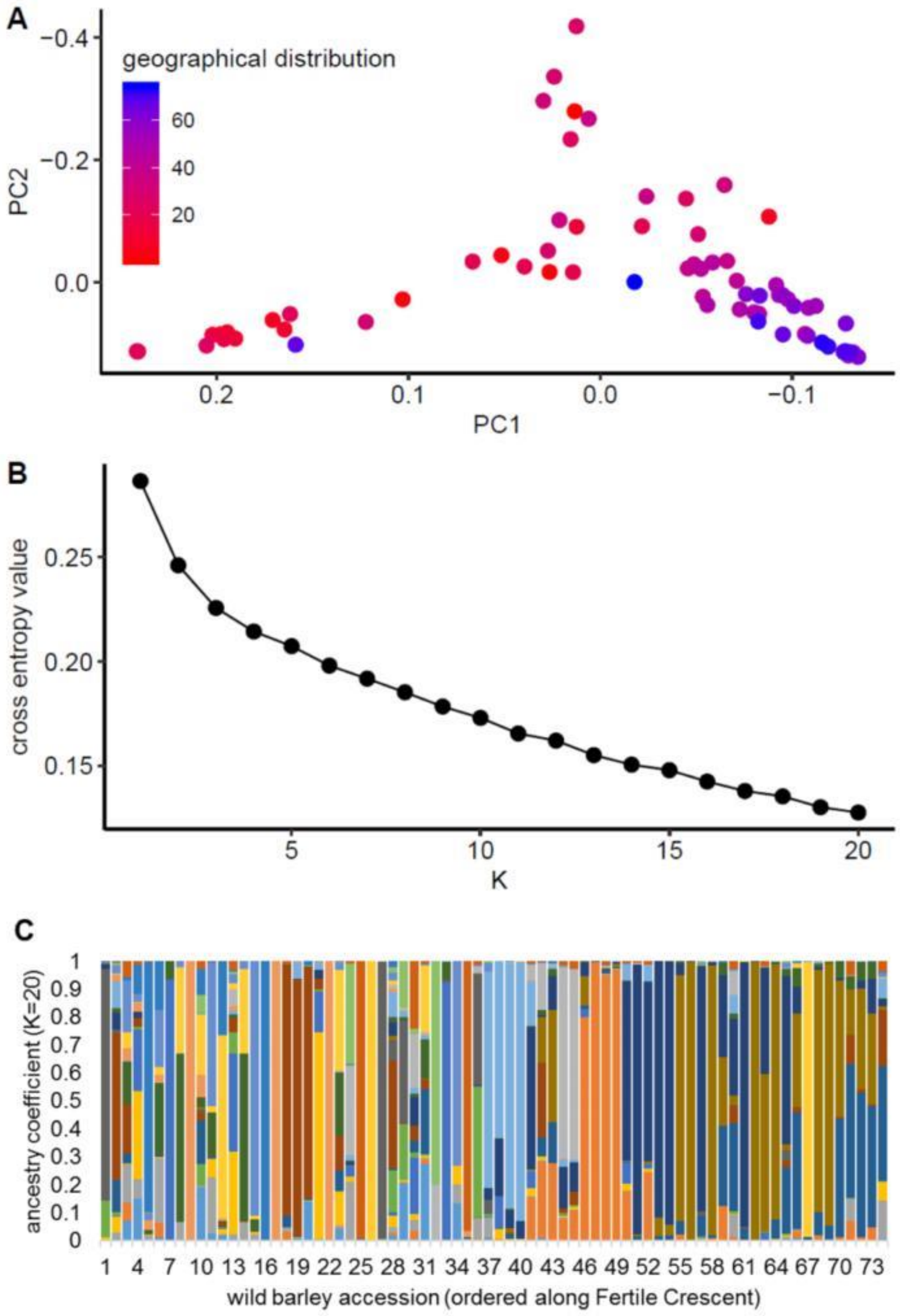

(A) Principal component analysis. Accessions (1 to 74) are colour coded according to their geographical distribution along the Fertile Crescent (e.g. 1 (red) = accession 1 of sub-population 1 in the south-west of the Fertile Crescent, and 74 (blue) = accession 74 of sub-population 55 in the south-east of the Fertile Crescent). (B) Estimation of cross-entropy criterion for different numbers of ancestral populations ranging from K=1 to K=20. (C) Ancestry coefficients of wild barley accessions (K=20). Accessions are ordered according to their geographical distribution along the Fertile Crescent. Colours represent K=20 ancestral populations.

Supplementary Fig. 4. Sub-population analysis for individual chromosomes.

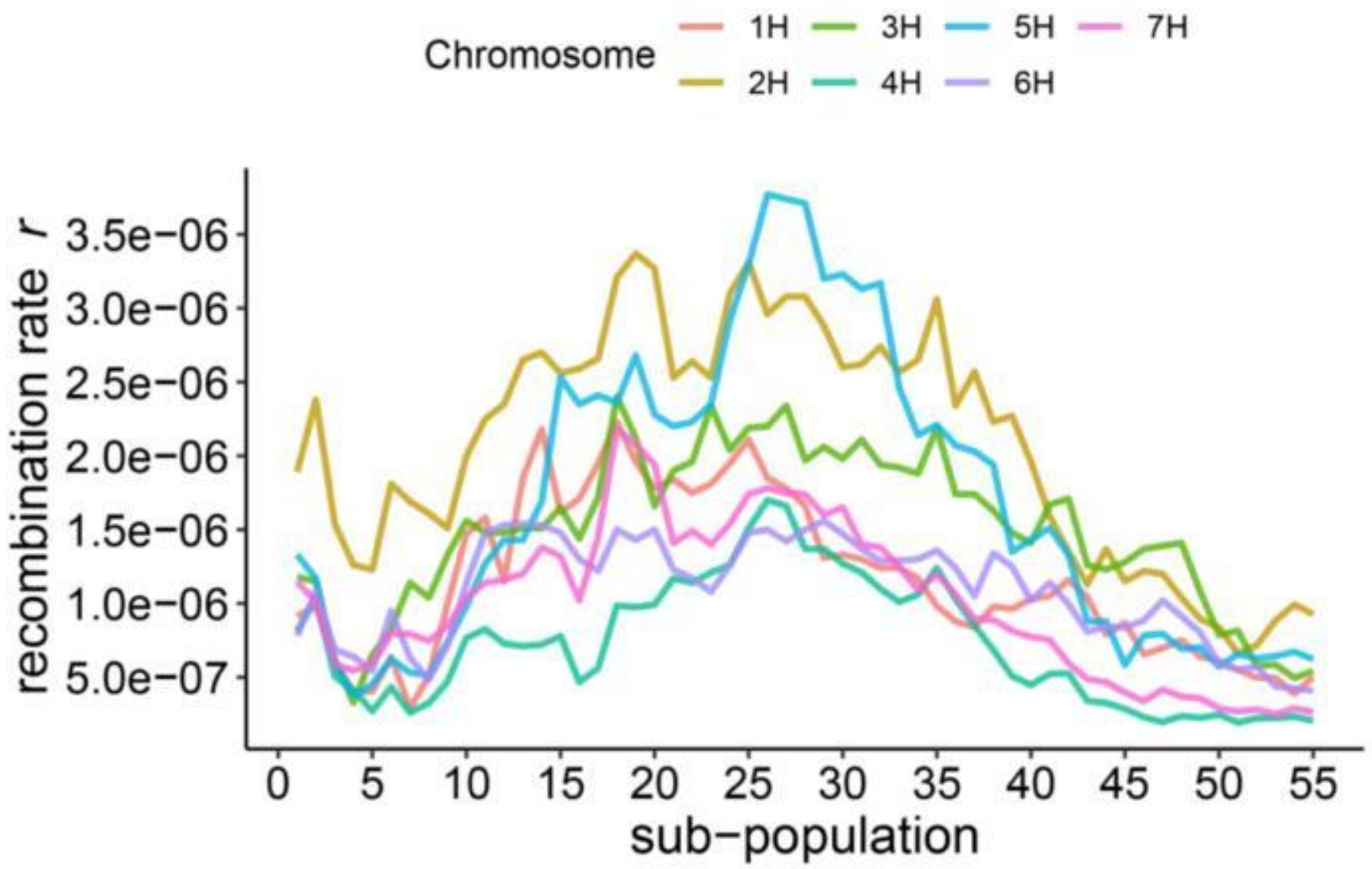

74 geo-referenced wild barley accessions are divided into 55 sub-populations of 20 accessions per sub-population according to a sliding window approach with an overlap of 1 accession. Sliding windows are moved along the geographical distribution of wild barley across the Fertile Crescent. Recombination rate *r* is shown for individual chromosomes coded in different colours.

Supplementary Fig. 5. Relationship between recombination rate and geographical distance within sub-populations.

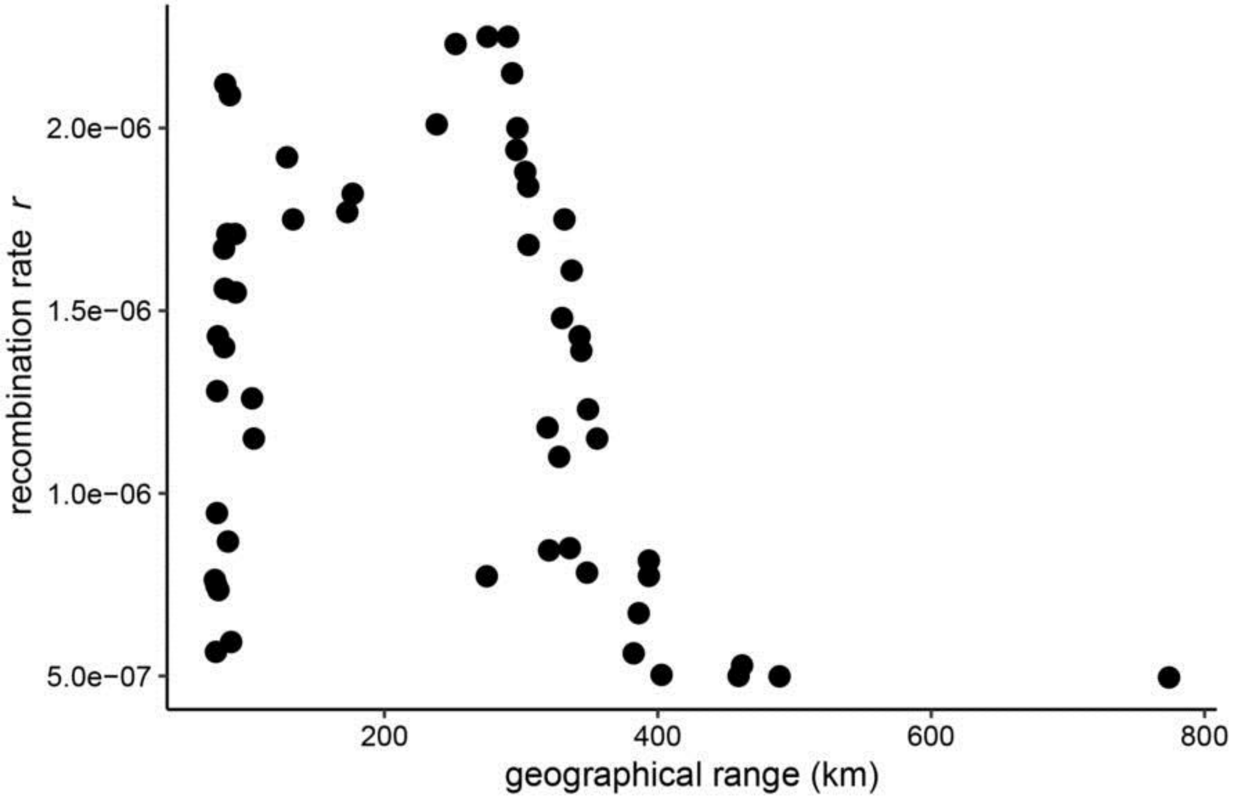

Recombination rate of the 55 sub-populations is plotted against the average geographical range $(((longitude_{max} - longitude_{min}) + (latitude_{max} - latitude_{min}) + (elevation_{max} - elevation_{min}))/3)$ within each sub-population.

Supplementary Fig. 6. Correlation between recombination rate and Last Glacial Maximum environmental conditions.

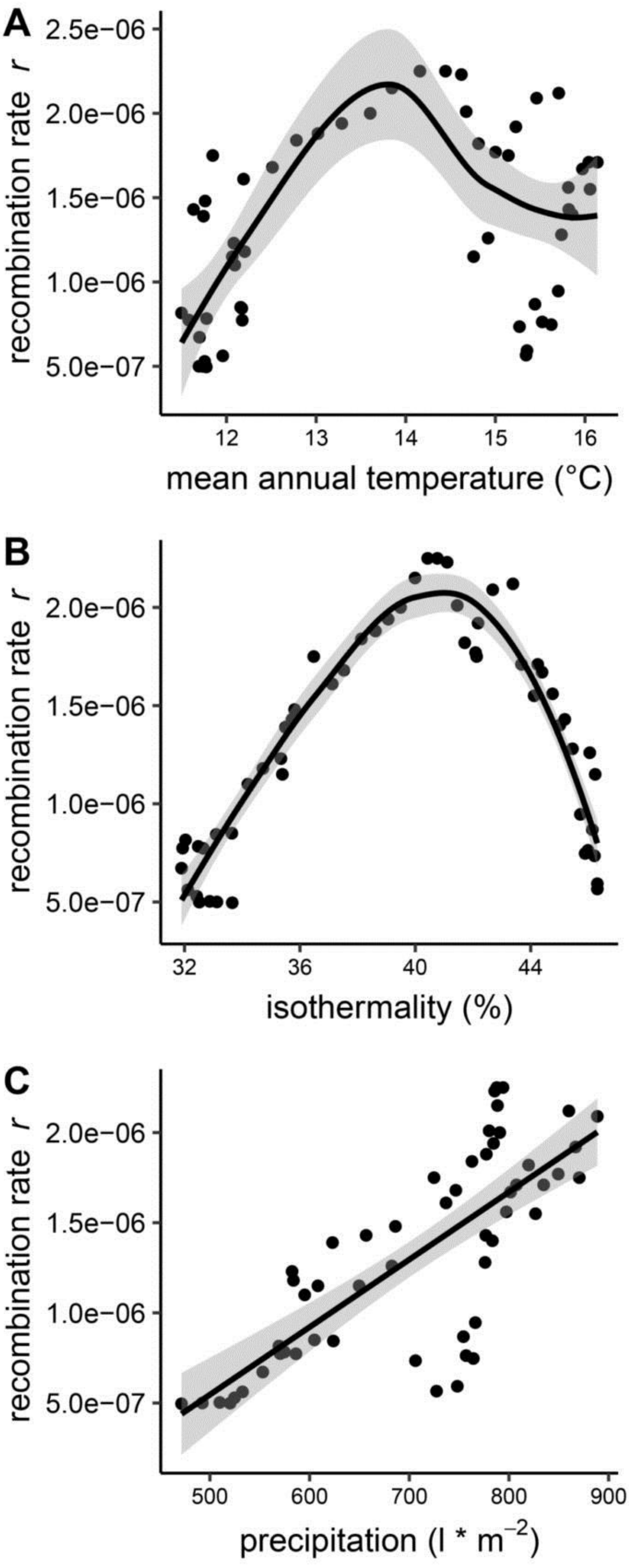

Correlation between recombination rate and (A) annual mean temperature, (B) isothermality, and (C) annual precipitation.

Supplementary Fig. 7. Correlation between recombination rate and Present environmental conditions.

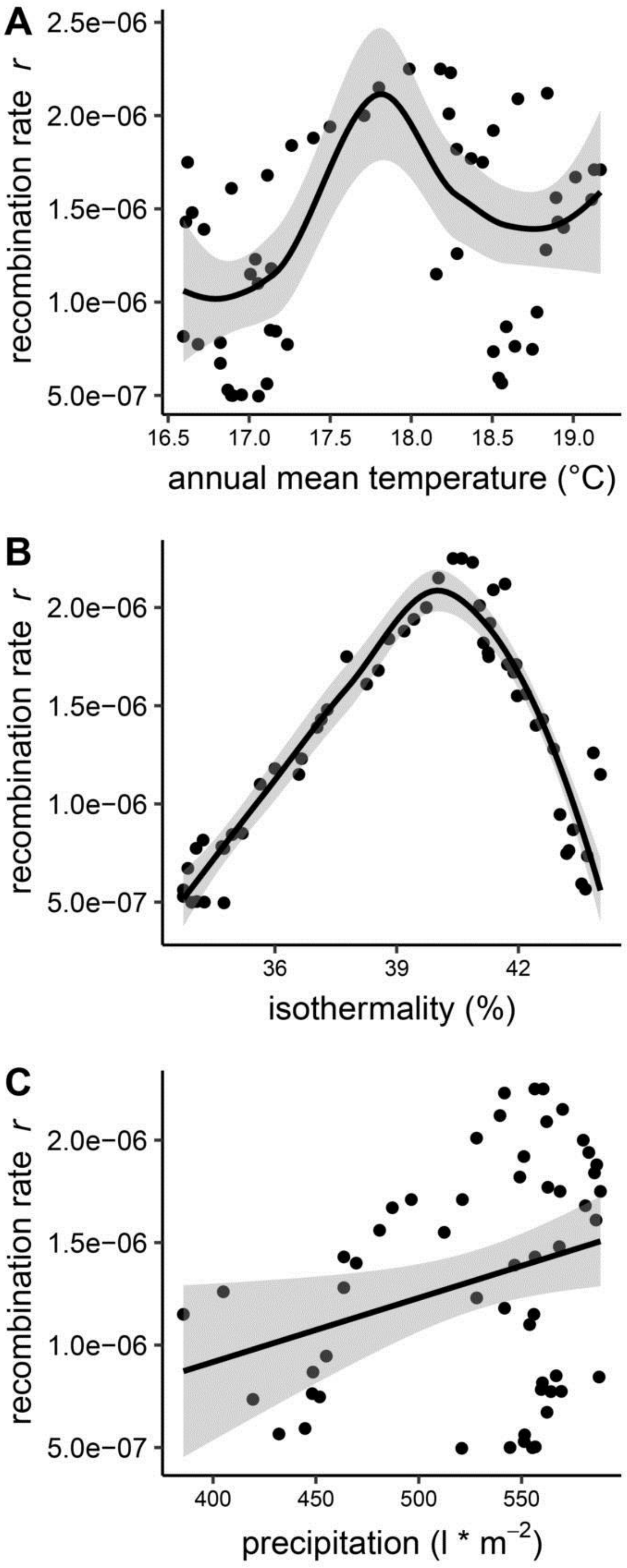

Correlation between recombination rate and (A) annual mean temperature, (B) isothermality, and (C) annual precipitation.

Supplementary Fig. 8. Randomization of sub-populations and correlation with environmental variables.

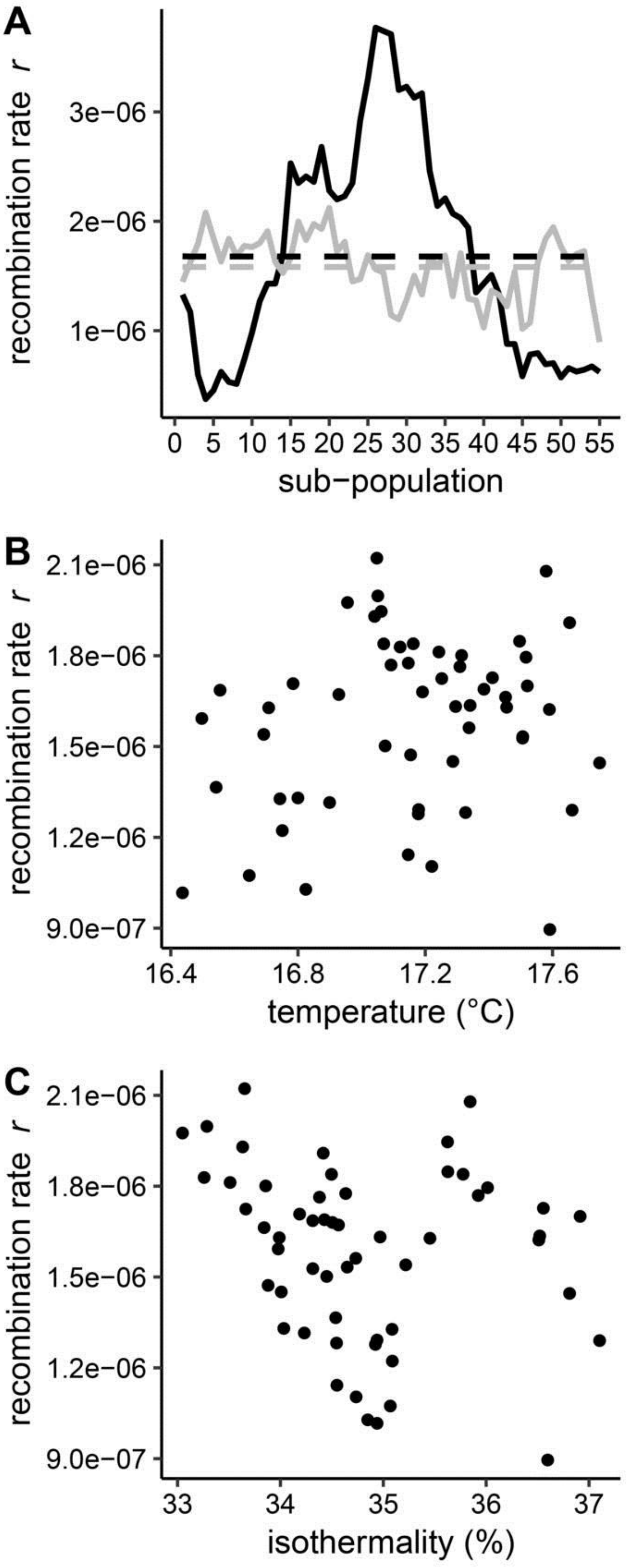

Wild barley accessions were randomly assigned to sub-populations, in contrast to assignment by geographical distribution, to test for a systematic bias in the sliding window approach. (A) Recombination rate estimated for chromosome 5H in geographically assigned sub-populations (solid black line, average shown as dashed black line) and randomly assigned sub-populations (gray line, average shown as dashed gray line). The average recombination rate in both approaches is similar, and variation between sub-populations is stronger if grouped according to geographical distribution. Recombination rate of randomly assigned sub-populations plotted against (B) annual mean temperature and (C) isothermality under Mid Holocene conditions.
